# Reverberation-Robust Self-Calibration and Synchronization of Distributed Microphone Arrays by Mitigating Heteroscedasticity and Outlier Occurrence in TDoA Measurements

**DOI:** 10.3390/s24010114

**Published:** 2023-12-25

**Authors:** Szymon Woźniak, Konrad Kowalczyk

**Affiliations:** Faculty of Computer Science, Electronics and Telecommunications, Institute of Electronics, AGH University of Kraków, al. Adama Mickiewicza 30, 30-059 Kraków, Poland; konrad.kowalczyk@agh.edu.pl

**Keywords:** distributed sensor networks, calibration, iterative reweighting, microphone array processing, robust estimation, self-localization, synchronization, localization, sensors

## Abstract

The network of distributed microphone arrays is usually established in an ad hoc manner; hence, network parameters such as the mutual positioning and rotation of the arrays, positions of sources, and synchronization of their recording onset times are initially unknown. In this article, we consider the problem of passively jointly self-calibrating and synchronizing distributed arrays in reverberant rooms. We use a typical two-step approach where, initially, the relative geometry of the network is estimated using Direction of Arrival (DoA) measurements. Subsequently, the absolute scale and synchronization parameters are estimated using Time Difference of Arrival (TDoA) measurements. This article presents methods to improve the robustness and accuracy of estimation of the absolute geometric scaling and synchronization parameters in reverberant conditions, in which TDoA measurements do not follow a normal distribution; furthermore, outliers often occur. To remedy these issues, we propose a Weighted Least Squares (WLS) estimator and schema for weighting the TDoA measurements to increase the estimation accuracy from heteroscedastic TDoA measurements. In addition, we propose an iterative reweighing algorithm with a binary weight to detect and reject TDoA outliers, which exploits the residuals of the parametric model in the least absolute value minimization. A numerical evaluation shows significant improvements in the proposed method over the state of the art in terms of the relative scaling error and mean absolute value of the synchronization parameters.

## 1. Introduction

Smart devices are often equipped with one or more microphones [1], which enable advanced audio signal processing [2], such as spatial filtering [3,4], source localization [5,6,7], and distributed signal enhancement [2]. When several devices with independent sampling clocks are distributed in space and interconnected using wireless data transmission, the setup is referred to as a Wireless Acoustic Sensor Network (WASN) [1]. Due to its ad hoc nature, calibration is usually required before any distributed processing can be performed [8].

Common approaches to self-localization in the WASN employ information about the distances between sensors and sources, which can be inferred from Time of Flight (ToF) measurements. In these approaches, the entire set of ToF measurements is processed to determine the positions of sensors and sources. For example, this can be achieved using the recently proposed iterative weighted least squares method [9], which is initialized with a multidimensional scaling technique. Another popular method is the so-called bilinear approach [10], which exploits matrix factorization through singular value decomposition and coordinate transformation. The latter approach also allows one to find solutions in a closed-form manner when certain geometrical conditions are met [11]. Alternatively, it is also possible to synchronize the network using ToF measurements, using low-rank alternating projections [12] or total least squares [13]. In practice, however, these methods require fully controlled sources (i.e., the knowledge of the source signal) to extract ToF measurements, which is usually impractical in realistic ad hoc network setups.

On the other hand, there exist much more practical approaches to the self-calibration of distributed microphone arrays that are based on Time Difference of Arrival (TDoA) and/or Direction of Arrival (DoA) measurements, which can be reliably detected [14] directly from signals observed at acoustic sensors without any knowledge of the source signals. In general, approaches with uncontrolled sources are referred to as passive self-localization. Self-localization based only on TDoA measurements usually exploits low-rank properties. Examples of methods that employ TDoA measurements include a three-step stratification process with rank constraints and factorization [15], a low-rank approximation using a pseudo-matrix of Time of Arrival (ToA)s [16], and a constrained total least squares approach [17]. Unfortunately, all of the aforementioned passive approaches are derived either for a specific number of sensors and sources, or the convergence of the method to the optimal solution is often troublesome. If multiple sensors are available within a single device, directional information about DoAs of the incoming waves impinging on such an array can also be incorporated into self-localization as additional spatial information. Joining the measurements of TDoA and DoA into a single cost function was proposed in [18], while the solution was found using iterative optimization methods. Since part of these measurements may be unreliable due to, for example, strong room reflections, some solutions to deemphasize unreliable measurements have been discussed in [19]. Recently, a method was proposed for splitting the cost function into multiple local cost functions, each assigned to a different node. Following this, distributed array calibration using the so-called damped Newtonian optimization was proposed in [20]. Another approach [21] consists of combining two types of objective functions, for orientation and time offset, respectively, with the aim of simultaneously estimating both these parameters in a distributed self-calibration and synchronization problem. Interesting topics of current research encompass solutions for scaling WASN [22], in which the weighted least squares algorithm is applied to find calibration parameters when a new node is added to the existing WASN. Another area of study involves using implicit representations of source positions in ToA-based self-calibration problems [23]. Similar to the aforementioned passive self-localization and synchronization, TDoA and DoA measurements have been jointly exploited in the self-localization of acoustic transceiver networks, such as in [24], and an attempt to split the centralized two-step cost function for geometry calibration of the acoustic transceiver network into local cost functions was presented in [25] using distributed Newton optimization. Note that the deployment of the latter approaches is, however, restricted to transceiver nodes only, i.e., each node of the network needs to contain both the microphone array and the loudspeaker.

In this article, we focus on two-step approaches for passive self-localization and synchronization of distributed microphone arrays. Initially, the relative geometry of the WASN (up to an arbitrary scaling factor) is estimated based only on the DoA measurement set using either an angular-based estimator [26,27,28,29,30], or a ray-based estimator [31]. The benefit of this approach is that, due to better convergence properties, it does not require an application of the Random Sample Consensus (RANSAC) [32] technique, which significantly increases the calibration speed. For details of the first step, i.e., relative geometry estimation of sources and distributed acoustic sensor arrays, the reader is referred to a short survey provided in [31]. The second step exploits TDoA measurements and the relative geometry estimated in the previous step to find the absolute geometry (i.e., the absolute positions of the sources and sensor arrays), which is achieved by estimating the so-called scaling factor, which scales up or down the overall relative geometry. Since the devices that create the WASN are usually not synchronized with each other, the TDoA measurements for independent devices will be biased. Therefore, the synchronization parameters of WASN should also be estimated in the second step [29]. We discuss in more detail several approaches for estimating the scaling factor value in further parts of the article.

In this work, we consider a scenario in which WASN is located in a reverberant room. In fact, this is a typical scenario in which microphone arrays are deployed in practice. However, although several studies present evaluations of self-calibration and synchronization in reverberant environments [18,27], the existing approaches are not designed to countermeasure reverberation, and this problem has so far rather been overlooked by the research community. Room reverberation is a well-known phenomenon [33] that occurs when a propagating wave is reflected from the surfaces encountered; therefore, the signal received by any microphone can be considered to be a mixture of multiple copies of the signal emitted by the sound source with different delay times and attenuations. As a result, the TDoAs measured from the signals captured in the reverberant room deteriorate significantly and do not follow a normal distribution. A significant deterioration in the TDoA measurements (from their true values) undoubtedly has a negative impact on any processing that is based on these measurements. In particular, any self-calibration procedure will suffer greatly since the measurements used for calibration are usually expected to be precise. Therefore, the problem of self-calibration and synchronization from measurements performed in reverberant acoustic conditions should be considered one of the main issues that this article aims to resolve. We distinguish two attributes of such measurements: firstly, heteroscedasticity and secondly, the presence of outliers, and subsequently, we propose an approach to effectively address both of these issues. Note that heteroscedasticity is a property of a set of random variables where the variances of each variable differ. In the context of this article, we assume that the variance of each measured time difference of arrival (TDoA) depends on the distances between the source and the microphones, which are randomly distributed in ambient space. Thus the main contribution of this article is a novel iterative algorithm for estimating the scaling factor and recording time onsets, using a heteroscedastic TDoA measurement set that also contains outliers. Additional contributions include a detailed experimental evaluation of the novel algorithm and a comparison with state-of-the-art methods.

The structure of this article is as follows. Section 2 provides a comprehensive formulation of the problem of passive self-localization and synchronization of the network of distributed microphone arrays. Section 3 discusses practical problems of absolute geometry estimation with TDoA measurements, and introduces a novel algorithm for robust estimation of the scaling factor and synchronization onset times. Major novelties include the weighted least squares estimator and an iterative reweighting scheme. Section 4 presents the results of the conducted numerical evaluations that show a comparison of the proposed method with the state-of-the-art approach under various acoustic conditions, including room reverberation. Finally, Section 5 concludes the article.

## 2. Problem Statement

Let us consider a WASN composed of *N*-independent devices distributed in a *D*-dimensional space (in practical scenarios, either two-dimensional or three-dimensional rooms are considered, i.e., D=2 or D=3), which captures *S* consecutive acoustic events. The position of the *i*th device, where i=1,⋯,N, is given by vector $n_{i}\in R^{D}$, while positions of all devices are grouped into the matrix $N={\left[n_{1},\;n_{2},\;\cdots ,\;n_{N}\right]}^{T}\in R^{N\times D}$. Each device is equipped with a microphone array that has a local geometrical structure known a priori. The orientation of the *i*th microphone array (the orientation of the microphone array is equivalent to the orientation of the device) located at position $n_{i}$ in the room is defined by vector $\theta_{i}\in R^{(D^{2}-D)/2}$, consisting of the Euler angles, which are further grouped into the matrix $\Theta ={\left[\theta_{1},\;\theta_{2},\;\cdots ,\;\theta_{N}\right]}^{T}\in R^{N\times (D^{2}-D)/2}$. The position of the *j*th acoustic event is denoted by vector $s_{j}\in R^{D}$ and positions of all the acoustic events form matrix $S={\left[s_{1},\;s_{2},\;\cdots ,\;s_{S}\right]}^{T}\in R^{S\times D}$. The basic geometric relation between the position and orientation of the *i*th device and the position of the *j*th acoustic event is defined by vector $p_{i,j}\in R^{D}$, which represents the position of the *j*th acoustic event with respect to the local position of the reference microphone of the *i*th device, which is given by the following equation

(1) $$p_{i,j}=R\;\left(\theta_{i}\right)\left(s_{j}-n_{i}\right),$$

where $R\;\left(\theta_{i}\right):R^{(D^{2}-D)/2}⟶R^{D\times D}$ is a function that generates the rotation matrix from Euler angles $\theta_{i}$.

**Figure 1:**
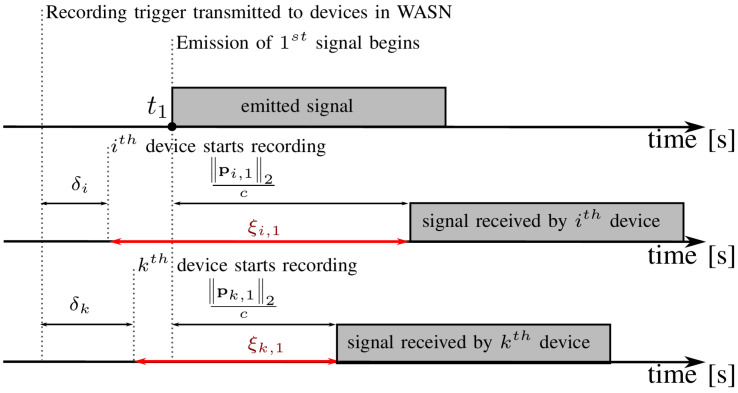
Timeline of events in the WASN when it begins to capture signals. Firstly a message triggering the recording is sent to all devices in the network. Each device begins to record the signal after receiving the message, but due to the latency in the network, individual devices start to record the signals related to the acoustic event at different points in time.

Since all devices in WASN are independent, we assume that there exists no precise synchronization of the starting points of audio capture between devices in the network. The time point at the beginning of the recording taken by the *i*th device is modeled as a real scalar value $\delta_{i}\in R$. The recording onset times of all devices that build WASN are collected in a single vector $\Delta =\left[\delta_{1},\delta_{2},\cdots ,\delta_{N}\right]\in R^{N}$. Finally, the emission time of the *j*th acoustic event is denoted as $t_{j}\in R$. Similar to the majority of self-calibration methods from the literature, in this work, we assume that there is no overlap in time between any pair of the source signals. As a result, due to the existence of different starting points of the signal capture by each distributed device, the ToA $\xi_{i,j}\in R$ of the direct sound of the *j*th acoustic event—as captured by the *i*th device—is additionally biased by $\delta_{i}$. Thus, ToA can be modeled as follows:

(2) $$\xi_{i,j}=\frac{\left\|{s_{j}-n_{i}}_{2}\right\|}{c}+t_{j}-\delta_{i},$$

where *c* is the speed of sound and ${\left\|\;\cdot \;\right\|}_{p}$ denotes the *p*-norm $L_{p}$. The timeline of the aforementioned events that occur when WASN starts to capture the microphone signals associated with an acoustic event is presented in Figure 1.

In this work, our aim is to estimate a set of unknown parameters N, Θ, Δ, S using only the quantities that can be measured passively, without knowing the precise time point when the acoustic event takes place (i.e., in our passive approach $t_{j}$ is an unknown parameter) and without the knowledge of the waveform of the acoustic signal that is emitted (which constitutes the so-called uncontrolled source). To this end, we exploit only the measurements of DoA and TDoA of unknown acoustic events. For the *j*th acoustic event, the DoA $d_{i,j}\in R^{D}$ at the *i*th device is modeled as

(3) $$d_{i,j}={\left\|p_{i,j}\right\|}_{2}^{-1}\;\;p_{i,j}.$$

In this work, DoAs are measured using intra-array signals, and the geometry relative up to the scaling factor γ, denoted as Θ, $\tilde{N}$, $\tilde{S}$ is estimated based on the set of DoA measurements [29]. To estimate the scale factor of geometry and synchronization offsets Δ, we exploit a set of measurable TDoAs between the *i*th and *k*th devices that capture the *j*th acoustic event, which can be modeled as

(4) $$\tau_{i,k}^{j}=\xi_{i,j}-\xi_{k,j}=\frac{{\left\|s_{j}-n_{i}\right\|}_{2}-{\left\|s_{j}-n_{k}\right\|}_{2}}{c}-\delta_{i,k}\;,$$

where $\delta_{i,k}=\delta_{i}-\delta_{j}$.

In this article, we propose a two-step Maximum Likelihood (ML) method for the self-localization and synchronization of distributed microphone arrays, consisting of the following processing steps. First, we find the relative geometry (i.e., Θ, $\tilde{N}$, and $\tilde{S}$), based on the measured DoAs using an ML approach that exploits the concept of half lines, known as rays [31]. We selected this approach because it is known to be highly robust toward random parameter initialization compared to many state-of-the-art approaches, as shown e.g., in  [31]. Next, we refine the relative geometry using the ML estimator derived using directional statistics with robustness incorporated against DoA measurement errors [30]. Finally, we perform the second step, which consists of the estimation of the scaling factor and synchronization offset times using an iterative method proposed in Section 3. As will be shown in experimental evaluations in Section 4, the proposed approach enables us to successfully increase the robustness against TDoA measurement errors, very effectively removing strong outliers.

## 3. Absolute Geometry Estimation and Synchronization

The relationship between the relative geometry given by matrices $\tilde{N},\tilde{S}$ and the absolute geometry given by matrices N,S can simply be defined as

(5a) $$N=\gamma \;\tilde{N},$$

(5b) $$S=\gamma \;\tilde{S},$$

where γ∈R is the scaling factor. This ambiguity in the relative geometry can be resolved by estimating the scaling factor, which can be achieved using the measured TDoAs. In the literature, we can distinguish between two approaches to the estimation of the scaling factor, intra-array approach which involves measurements between the signals of microphones within a single array, and the inter-array approach, involving measurements between the signals of microphones from different arrays. Usually, only the signal of a single (reference) microphone from each array is used to mitigate the computational load) TDoAs.

An estimation of the scaling factor using intra-array TDoAs was presented in [28] for circular microphone arrays. The main advantage of this approach is the lack of bias caused by synchronization offsets in measured TDoAs since all measurements applied for the estimation of the scale parameter originate from a single device. Unfortunately, this approach requires precise TDoA measurements due to the high sensitivity of localization to measurement errors for acoustic sources located in the far field of the array. Thus, in practice, this approach is limited to scenarios where sources are located in close proximity to the arrays and, thus, WASN is limited to be rather small in size (e.g., a few devices, such as smartphones lying on a desk).

Those limitations do not affect the approaches based on inter-array TDoAs [26,27,29] since the magnitude of those measurements is usually greater by at least one order. In addition, the distribution of the positions of acoustic events and microphone arrays is much more similar. Consequently, the issue of sources being in the far field for the entire WASN (i.e., for every array simultaneously) is usually not encountered. This approach was initially used in [26,27], which exploits the known a priori relative geometry of the WASN and, hence, the estimation of the scaling factor usually involves just one or several individual TDoAs. For more realistic cases, where WASN is not synchronized prior to deployment, a similar strategy was used in [29] to derive the Least Squares (LS) estimator that, based on all measured TDoAs, jointly estimates the scaling factor of the relative geometry and the synchronization offset times.

**Figure 2:**
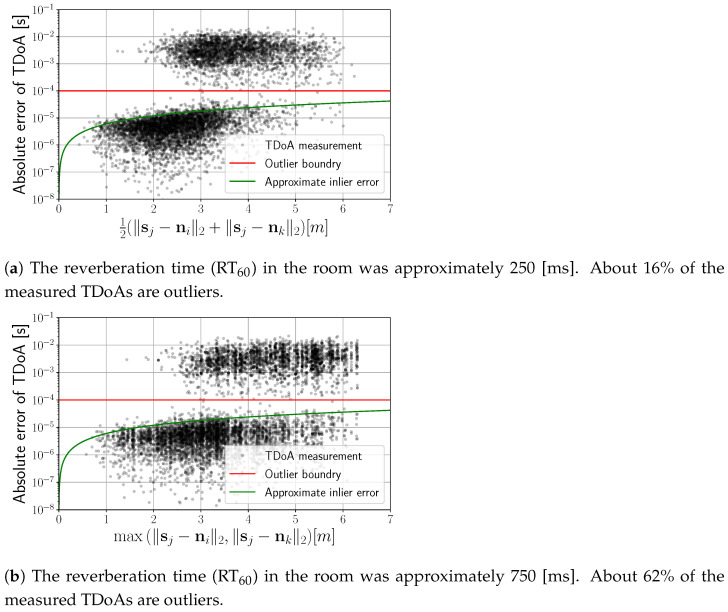
Distributions of the absolute inter-array time difference of arrival measurement error as a function of (**a**) the average distance between the position of the source and the positions of the microphones in the pair and (**b**) the maximum distance between the position of the source and the position of the microphones. TDoA measurements were estimated for randomly placed N=25 microphones and S=25 acoustic events generated by short speech utterances emitted at random positions in the reverberant room of 7×5×3 [m] size, and the self-noise of the microphones was −60 [dB]. The red line denotes the outlier boundary for the TDoA measurement error, which exceeds 10 [ms].

In addition to the synchronization offset bias, the TDoA measurements are affected by a phenomenon known as reverberation, which significantly obstructs the accuracy of those measurements. Figure 2 shows example distributions of absolute measurement errors between the true and inferred TDoA estimates in terms of the distance between the position of the source and the position of the microphone pair used to estimate a particular TDoA. We can observe two straightforward relations between the magnitude of the error and the distance. The first is a steady increase in the measurement error observed for an increasing distance, and the second is a significant probability increase of the occurrence of outliers when acoustic sources are farther away than a certain threshold distance. Both of those relationships are associated with a decreasing power of the signal that propagates over the direct path. The deterioration of the correlation between the received microphone signals is caused by the low power of the direct propagation path component, compared with the power of other propagation paths related to wave reflections at boundaries in the room. For a certain threshold distance, late reverberation starts to become a dominant component in the received signals (which yields a low direct-to-reverberant ratio), significantly increasing the probability of the occurrence of outliers among the estimated TDoAs.

Section 3.1 introduces a weighted LS estimator for the scaling factor and synchronization offsets; it attempts to mitigate the effect of an increasing TDoA measurement error at longer distances. In Section 3.2, we propose a new robust estimator of the scaling factor and synchronization offsets based on iterative reweighing with binary weights.

### 3.1. Weighted Least Squares for Heteroscedastic Measurements

The estimator for the scaling factor and synchronization offsets presented in [29] could be seen as a nonlinear ML estimator [16,34] with linear constraints on the microphone and source positions. According to the Gauss–Markov theorem, this estimator is efficient only for a homoscedastic set of TDoA measurements. In practice, the variance of the estimated TDoAs between the source and two microphones is not constant and strongly depends on the acoustical conditions of the environment. As a result, the TDoA measurements are actually heteroscedastic. Given this notion, we introduce here a modified version of the LS estimator described in [29], which takes into account the non-constant variability of each measured TDoA, which can be written as

(6) $${\hat{\gamma }}^{LS},{\hat{\Delta }}^{LS},\hat{N},\hat{S}=\underset{\Delta ,\gamma ,N,S}{\mathrm{argmin}}\sum_{j=1}^{S}\sum_{i=1}^{N}\sum_{k=1}^{N}w_{i,k}^{j}{\left(\tau_{i,k}^{j}-{\overset{ˇ}{\tau }}_{i,k}^{j}\right)}^{2}\;\mathrm{subject}\;\mathrm{to}\;N=\gamma \tilde{N}\wedge S=\gamma \tilde{S},$$

where $w_{i,k}^{j}\in R$ is a weighting coefficient for each estimated value of ${\overset{ˇ}{\tau }}_{i,k}^{j}$. As shown in [29], it is possible to reformulate problem (6) as an unconstrained problem. To this end, the modeled value of TDoA, denoted by $\tau_{i,k}^{j}$, can be expressed as

(7) $$\tau_{i,k}^{j}=\gamma \;{\tilde{\tau }}_{i,k}^{j}-\delta_{i,k},$$

where ${\tilde{\tau }}_{i,k}^{j}$ is the relative TDoA, computed based on the known relative geometry in the following manner:

(8) $${\tilde{\tau }}_{i,k}^{j}=c^{-1}\left(\|{{\tilde{s}}_{j}-{\tilde{n}}_{i}\|}_{2}-\|{{\tilde{s}}_{j}-{\tilde{n}}_{k}\|}_{2}\right).$$

As a result of this substitution, a constrained problem (6) is reformulated into the weighted linear LS problem given by

(9) $${\hat{\gamma }}^{LS},{\hat{\Delta }}^{LS}=\underset{\gamma ,\Delta }{\mathrm{argmin}}\sum_{j=1}^{S}\sum_{i=1}^{N}\sum_{k=1}^{N}w_{i,k}^{j}{\left(\gamma {\tilde{\tau }}_{i,k}^{j}-\delta_{i}+\delta_{j}-{\overset{ˇ}{\tau }}_{i,k}^{j}\right)}_{2}^{2},$$

where ${\hat{\gamma }}^{LS},{\hat{\Delta }}^{LS}$ denotes the weighted least squares solution [35]. Problem (9) consists of solving an overdetermined system of $SN^{2}$ linear equations with the following unknown variables $\gamma ,\delta_{1},\delta_{2},\delta_{3},\cdots ,\delta_{N}$, by minimizing the value of the squared residuals. To control the impact of each equation (i.e., residual) on the solution, the weights $w_{i,k}^{j}$ for each residual are introduced. The matrix form of problem (9) is given as

(10) $${\hat{q}}^{LS}=\underset{q}{\mathrm{argmin}}{\left\|Aq-\overset{ˇ}{\tau }\right\|}_{2}^{2},$$

where $A\in R^{SN^{2}\times N}$ denotes the matrix of weighted coefficients of the system of linear equations, $\overset{ˇ}{\tau }\in R^{SN^{2}}$ denotes the vector of weighted constant terms, and q denotes the unknown variables of the system. Since the solution ${\hat{\Delta }}^{LS}$ is invariant to translation, we constrain the value of ${\hat{\delta }}_{1}^{LS}$ to be 0, as suggested in [29]. The solution to the problem given by (10) can be conventionally computed in closed form using the Moore–Penrose pseudoinverse of matrix A, and it is given as

(11) $${\hat{q}}^{LS}=A^{†}\overset{ˇ}{\tau },$$

where matrix A and vector $\overset{ˇ}{\tau }$ can be built using Algorithm 1, $\left(\;\cdot \;\right){}^{†}$ denotes a Moore–Penrose pseudo-inverse of the matrix, and vector ${\hat{q}}^{LS}={\left[{\hat{\gamma }}^{LS},{\hat{\delta }}_{2}^{LS},{\hat{\delta }}_{3}^{LS},\cdots ,{\hat{\delta }}_{N}^{LS}\right]}^{T}\in R^{N}$ contains optimal (in the least squares sense) values of the scaling factor and synchronization offsets.    
**Algorithm 1:** Synthesis of the design matrix A and measurement vector $\tilde{\tau }$. It constitutes a modified version of the algorithm initially presented in [29]
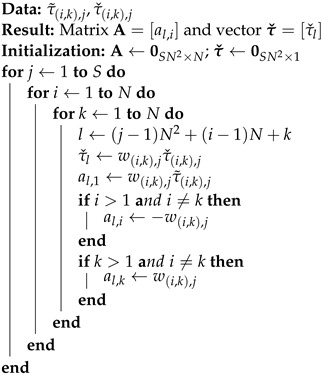


To set the weights for the problem given by (6), we exploit the a priori known relative geometry of WASN and domain knowledge. As noted earlier (cf. Figure 2), the variance of the TDoA measurements depends on the distance between the sound source and the microphones that received this signal. This is a well-known relation that results from the occurrence of reverberation in the room [33,36]. Since the absolute distances between the sources and microphones are unknown, we exploit the relative distances that can be calculated using the relative geometry $\tilde{N}$, $\tilde{S}$, as the latent variable that indirectly indicates the variance of the measured ${\overset{ˇ}{\tau }}_{(i,k),j}$. We propose using the maximum distance between the source and the microphones in the relative geometry $\phi_{(i,k),j}^{\max}\in R$, which can be written as

(12) $$\phi_{(i,k),j}^{\max}=\max\;\left(\|{{\tilde{s}}_{j}-{\tilde{n}}_{i}\|}_{2},\|{{\tilde{s}}_{j}-{\tilde{n}}_{k}\|}_{2}\right),$$

or an average distance $\phi_{(i,k),j}^{\mathrm{avg}}\in R$ given as

(13) $$\phi_{(i,k),j}^{\mathrm{avg}}=\frac{{\left\|{\tilde{s}}_{j}-{\tilde{n}}_{i}\right\|}_{2}+{\left\|{\tilde{s}}_{j}-{\tilde{n}}_{k}\right\|}_{2}}{2}.$$

As shown in Figure 2, both of these variables capture the heteroscedasticity of the TDoA measurements. Finally, to take into account the heteroscedasticity of the measured TDoA, we propose to set the weights $w_{(i,k),j}$ of the LS problem (6) to scalar values $d_{(i,k),j}\in R$, computed in the following manner:

(14) $$d_{(i,k),j}={\left(1-\frac{\phi_{(i,k),j}}{\max_{x,y,z}\;\phi_{(x,y),z}}\right)}^{\beta },$$

where parameter β∈R controls the steepness of the transfer function, and $\phi_{(i,k),j}$ is either set as $\phi_{(i,k),j}^{\max}$ or $\phi_{(i,k),j}^{\mathrm{avg}}$.

### 3.2. Robust Estimation with Iterative TDoA Reweighing

The main disadvantage of the LS estimator given by (9) is its high sensitivity to outliers in the TDoA measurement set. As a result, the solution given by (11) will usually be significantly deteriorated by outliers, which can reduce the performance of the estimator in practical deployment in reverberant rooms. As mentioned earlier, the probability of the occurrence of outliers substantially increases when the distance between the source and one of the microphones exceeds a certain threshold value. Unfortunately, since we only operate on the relative distances, and conditions of the acoustical scene are usually also unknown, in this work, we do not exploit this prior, as proposed with the heteroscedastic measurement in Section 3.1.

To mitigate the impact of outliers, we propose using an iterative reweighing algorithm with binary weights for each TDoA measurement, which takes advantage of the minimization of the Least Absolute (LA) values. The LA estimation is known to be more robust than the LS estimation to the outliers due to the employed norm $L_{1}$ instead of the norm $L_{2}$ for the residuals, which places less emphasis on large residuals, thereby achieving an increase in robustness toward the outliers.

The problem of jointly estimating the scaling factor and synchronization offset parameters, along with the LA value can be written as

(15) $${\hat{\gamma }}^{L_{1}},{\hat{\Delta }}^{L_{1}}=\underset{\gamma ,\Delta }{\mathrm{argmin}}\;\;f_{L_{1}}\left(\gamma ,\Delta \right),$$

where ${\hat{\gamma }}^{L_{1}}$, ${\hat{\Delta }}^{L_{1}}$ are the estimated optimum parameters, and $f_{L_{1}}\left(\gamma ,\Delta \right)\in R$ is an objective function, defined as

(16) $$f_{L_{1}}\left(\gamma ,\Delta \right)=\sum_{j=1}^{S}\sum_{i=1}^{N}\sum_{k=1}^{N}w_{i,k}^{j}{\left\|\gamma \;{\tilde{\tau }}_{i,k}^{j}-\delta_{i,k}-{\overset{ˇ}{\tau }}_{i,k}^{j}\right\|}_{1}.$$

Unlike (9), the analytical (i.e., closed-form) solution to the problem given by (15) with (16) does not exist and it is usually found with iterative methods such as simplex-based approaches [37], the direct descent approach [38], or the maximum likelihood estimation [39]. In this work, we solve problem (15) by minimizing an objective function (16) with the quasi-Newton optimization method, known as L-BFGS [40,41,42], which exploits the gradient

(17) $$\nabla f_{L_{1}}={\left[\frac{\partial f_{L_{1}}}{\partial \gamma },\frac{\partial f_{L_{1}}}{\partial \delta_{1}},\frac{\partial f_{L_{1}}}{\partial \delta_{2}},\;\cdots ,\frac{\partial f_{L_{1}}}{\partial \delta_{N}}\right]}^{T}\in R^{N}$$

of the objective function $f_{L_{1}}$ from (16), with vector elements, respectively, given by

(18a) $$\frac{\partial f_{L_{1}}}{\partial \gamma }=\sum_{j=1}^{S}\sum_{i=1}^{N}\sum_{k=1}^{N}w_{i,k}^{j}\;r_{i,k}^{j}{\tilde{\tau }}_{i,k}^{j}\;,$$

(18b) $$\frac{\partial f_{L_{1}}}{\partial \delta_{z}}=\sum_{j=1}^{S}\left(\sum_{i\neq z}^{N}w_{i,z}^{j}r_{i,z}^{j}-\sum_{k\neq z}^{N}w_{z,k}^{j}r_{z,k}^{j}\right),$$

(18c) $$r_{i,k}^{j}=\mathrm{sgn}\left(\gamma \;{\tilde{\tau }}_{i,k}^{j}-\delta_{i,k}-{\overset{ˇ}{\tau }}_{i,k}^{j}\right)\;,$$

where sgn(x) is the sign function . Since the objective function $f_{L_{1}}$ is not differentiable in the entire domain, due to the fact that the sign function is not defined for 0 value, we set sgn(0) to be equal to zero, i.e., sgn(0)=0. Consequently, the gradient $\nabla f_{L_{1}}$ for non-differentiable points will be transformed into a subgradient [42,43]. Since the objective function $f_{L_{1}}$ is, in general, convex, the proposed quasi-Newton method should tend to converge to global minima, resulting in the optimal solution [42]. In practice, we did not encounter any problems with the convergence of the method to the optimal solution, but we found that random initialization of estimated parameters might result in a large number of iterations required to reach convergence. To reduce the number of iterations required for convergence to the optimum solution, we propose using the least squares solution ${\hat{\gamma }}^{LS}$, ${\hat{\Delta }}^{LS}$, which can be calculated according to (11), as a starting point for the proposed optimization method. We also investigated the modified $L_{1}$ loss function, such as the Huber loss function [44] or pseudo-Huber loss function [45] for the objective function (16), but did not observe an improvement in precision with such modifications.

**Figure 3:**
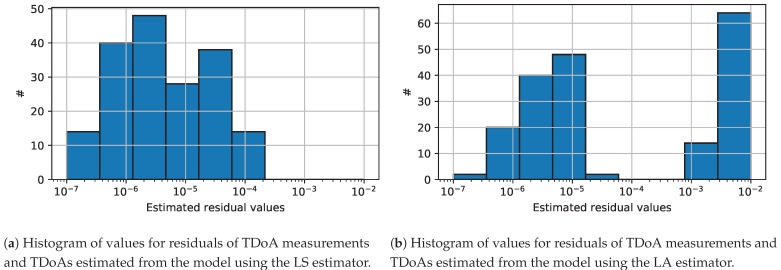
Comparison of the histogram of residuals for the scaling factor and synchronization offset parameters obtained by (**a**) the LS and (**b**) the LA estimators.

**Figure 4:**
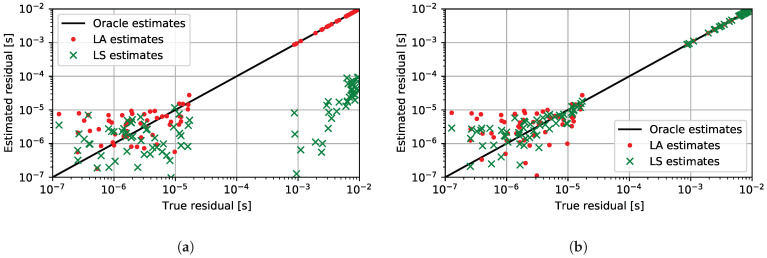
Comparison of the residual distribution for the scaling factor and synchronization offset parameters obtained by different estimators. Each individual point on the graphs represents the residual between the measured value and the TDoA value estimated by the parametric model. The axis denoted as *the true residual* represents the value of the residual for the ground truth parameters. Axis denoted as the *estimated residual* represents the value of the residual for the estimated parameters. The residuals for the oracle estimator would be on the black line. Points above the black line are overestimated. On the other hand, points under the black line are underestimated. Red dots denote the residuals that resulted from the LA estimation and green crosses denote the residuals that resulted from the LS estimation. (**a**) Comparison of residuals for LA and LS estimation methods without any weighting, i.e., all weights $w_{i,k}^{j}$ were set to 1. (**b**) Comparison of residuals for the LA and LS estimation methods after five iterations of the proposed reweighing method with binary weights.

The second interesting property of the LA estimator is that its solution could be exploited for the detection of outliers. To investigate this property, we analyze the distribution of residuals for the LA solution and compare it with the distribution of residuals for the LS solution, when the TDoA measurements contain outliers. The histograms of the residuals of the TDoA measurements for both solutions are presented in Figure 3, e.g., a simulated distributed scenario. As can be seen in Figure 3a, the residuals of the solution for the LS estimator yield a distribution that is strongly concentrated around some mean value, and the shape of the distribution resembles the Gaussian distribution. The characteristic of this distribution precludes any successful analysis with the purpose of detecting outliers. On the other hand, the residual distribution of the LA estimator presented in Figure 3b exhibits a clear separation between two groups of residuals, which vary in residual value. In the following, we further investigate the relationship between the residuals for the estimated parameters and the true residuals for the ground-truth parameters. These example relationships are presented in Figure 4. Based on the value of the *true residual*, we can reliably distinguish which measurement belongs to the outliers, i.e., a measurement with a true residual value greater than 1 × 10^−4^ , and which one belongs to the inliers, i.e., a measurement with a true residual value lower than 1 × 10^−4^ . If we closely analyze Figure 4a, then there is a substantial separation distance between the residual of outliers and the residuals of inliers on the true residual axis for both LA and LS estimators, which we expected. In the case of the LA estimator, the substantial separation distance can be observed as well for the *estimated residual* axis that correctly separates outliers from inliers. Furthermore, the residual values of the estimated parameters are much more correlated with the values of the true residuals, as their distribution is more concentrated around the oracle estimator line, cf. Figure 4.

In general, the separation distance between outliers and inliers of the residuals for the LA estimates depends on the unknown acoustic conditions and noise. The residual distribution for the unweighted LS estimator is generally underestimated, especially for outliers, cf. Figure 4a. From our observations over a substantial number of experiments, the above remarks on distributions of the residuals for LA and LS estimators hold with good approximation for varying acoustic conditions. The conclusion of this analysis is that, using residuals obtained from parameters estimated with LA value minimization, we can detect outliers by finding two clusters with small and large residuals. In general, this kind of problem can be classified as unsupervised learning, where we need to find those two clusters of residuals. For example, it could be successfully solved with a basic clustering algorithm, such as k-mean clustering [46]. Instead, to reduce the computational complexity of the method, we propose to apply a thresholding algorithm instead. Given the weights and the optimal solution for the LA value minimization, i.e., $\gamma^{L_{1}}$ and $\Delta^{L_{1}}$, the value of residual $r_{(i,k),j}^{L_{1}}\in R$ is computed as

(19) $$r_{(i,k),j}^{L_{1}}=w_{i,k}^{j}{\left\|{\hat{\gamma }}^{L_{1}}{\tilde{\tau }}_{(i,k),j}-{\hat{\delta }}_{i}^{L_{1}}+{\hat{\delta }}_{k}^{L_{1}}-{\overset{ˇ}{\tau }}_{(i,k),j}\right\|}_{1}\;.$$

Furthermore, based on the computed residuals, the two basic thresholding parameters are estimated, namely the mean value $\mu_{\tau }\in R$, given as

(20) $$\mu_{\tau }=\frac{2}{SN(N-1)}\sum_{j=1}^{S}\sum_{i=1}^{N}\sum_{k=i+1}^{N}r_{(i,k),j}^{L_{1}}\;,$$

and the standard deviation $\sigma_{\tau }\in R$, given as

(21) $$\sigma_{\tau }=\sqrt{\frac{2}{SN(N-1)-2}\sum_{j=1}^{S}\sum_{i=1}^{N}\sum_{k=i+1}^{N}{\left(r_{(i,k),j}^{L_{1}}-\mu_{\tau }\right)}^{2}}\;.$$

Finally, based on those two parameters and the residual for each measured TDoA, we propose to determine a binary weight $b_{(i,k),j}\in \left\{0,1\right\}$ using the following thresholding rule:

(22) $$b_{(i,k),j}=\left\{\begin{array}{cc} 1 & \;\mathrm{if}\;\left(r_{(i,k),j}^{L_{1}}-\mu_{\tau }\right)<\eta_{r}\sigma_{\tau }\\ 0 & \;\mathrm{otherwise} \end{array}\right.\;,$$

where $\eta_{r}$ is a tuning parameter for the rth iteration of the algorithm.

In each iteration, our aim is to change the tuning parameter in a manner, where $\eta_{r+1}>\eta_{r}$. The change in the value of the tuning parameter $\eta_{r}$, depending on the iteration, is motivated as follows. Initially, we aim to reject as many potential outliers as possible, even if a large number of inliers is rejected as well. As a result, the estimated parameters give a roughly better result, which translates to better outlier detection in the next iteration. Better outlier detection due to more correct parameter estimation allows one to relax the threshold (controlled by the tuning parameter), and as a result, increase the number of inliers. We advise starting with $\eta_{1}=1$ and gradually increasing it with a step of 0.5. Finally, our algorithm should exploit both types of weights, namely the aforementioned binary weight and the weight from the LS problem. Therefore, we proposed that the new weights $w_{(i,k),j}$ for each TDoA measurement are updated based on the computed binary weights $b_{(i,k),j}$ and the weights $d_{(i,k),j}$ proposed in the previous section $d_{(i,k),j}$, which is achieved by using the following relation:

(23) $$w_{(i,k),j}=d_{(i,k),j}b_{(i,k),j}\;.$$

With the use of the newly computed weights, the LS solution given by (11) is found and forms the final solution. The LS solution is chosen to calculate the final result since, in theory, it is considered the best linear unbiased estimator. If the assumed number of iterations is not completed, the LS solution is used as initialization for the LA estimator in the next iteration.

In general, the proposed iterative reweighing approach to the robust estimation of the scaling factor and the recording onset time is summarized in Algorithm 2. Example distributions of residuals of estimators LA and LS after five iterations of the proposed method can be seen in Figure 4b. A significant improvement in the residual distribution for the LS residuals was achieved by excluding outliers with proper weighting. The evaluation of the proposed method is presented in detail in Section 4.
**Algorithm 2:** The proposed iterative reweighing algorithm to the robust estimation of the scaling factor and recording onset time
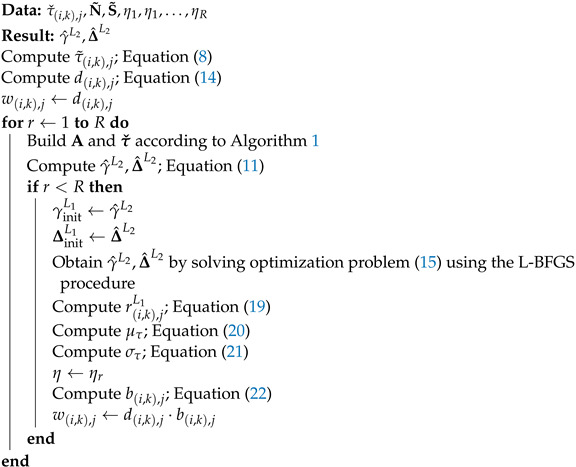


## 4. Experimental Evaluation

In this section, the proposed robust estimator of the scaling factor and recording onset times is evaluated. As a baseline method for performance comparison, we use an unweighted linear LS estimator proposed in [29]. Unless otherwise stated, all experiments are conducted in a three-dimensional (D=3) room of size 10×10×3 [m], with five tetrahedral microphone arrays (N=5) and ten acoustic sources (S=10). To obtain significant statistical results, in all experiments, we perform 1000 trials (K=1000) for each test point. For evaluation purposes, in each experimental trial, both microphone arrays and sources are placed randomly within the room with a uniform distribution throughout the room volume. For each array, the recording onset times are sampled from a uniform distribution with an interval of 1 to 100 [ms].

Since the evaluated method requires information about the relative geometry of WASN, we employ an approach presented in [31] to estimate it from DoA measurements. To estimate the DoA and TDoA measurements from the raw microphone signals, we use the well-known Steered Response Power with Phase Transform (SRP-PHAT) [47,48] and the Generalized Cross-Correlation with Phase Transform (GCC-PHAT) [49], respectively. The microphone signals are synthesized at a sampling frequency of 16,000 [Hz], by convolving room impulse responses (RIRs) with short (about 3 s long) speech segments. The room impulse response, for each pair of the microphone and source positions, is generated with the image source method [50]. Finally, white noise is added to the convoluted signal to simulate the microphone’s self-noise at the signal-to-noise ratio (SNR) level of around −60[dB]. Since the proposed method concentrates on the second optimization step, we aim to mitigate the impact of the relative geometry estimator on the presented results. To this end, if not stated differently, the DoA measurements are estimated from speech signals convolved with the RIR that contains only the direct propagation path between the source and the microphone, with added microphone noise. In this case, the variability of the DoA estimates does not depend on reverberation but it still deteriorates by microphone noise.

**Figure 5:**
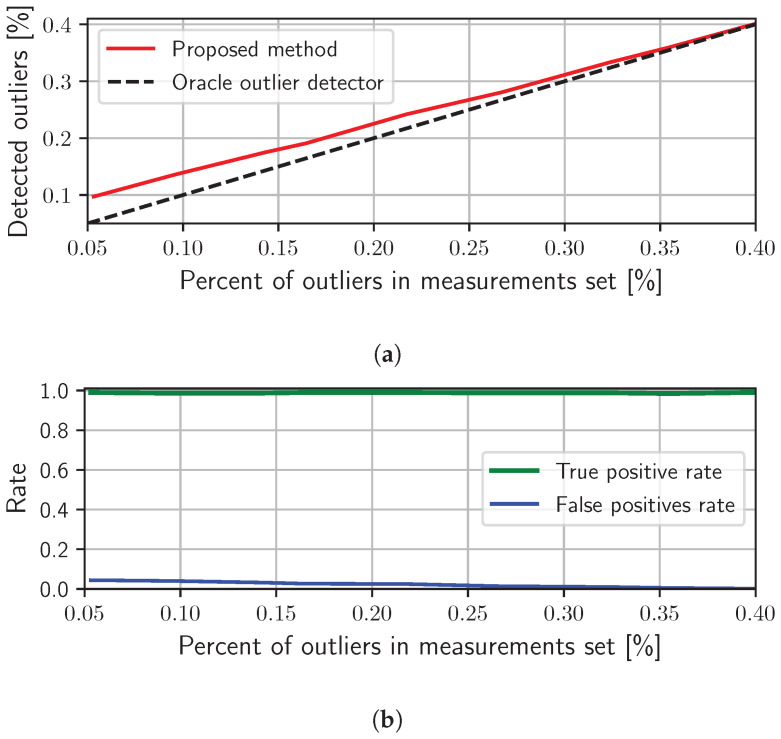
Evaluation of the proposed method in the context of outlier detection for a controlled number of outliers in the TDoA measurements set. (**a**) Presents the dependency between the number of outliers in the measurements and the number of outliers detected with the proposed method. (**b**) Shows the rates of correctly detected outliers (true positive rate) and the rates of incorrectly marked inliers as outliers (false positive rate) by the proposed method.

In the first experiment, we aim to evaluate the ability of the proposed iterative reweighing approach to detect outliers in the TDoA measurement set by properly setting the binary weight $b_{(i,k),j}$ to the value of 0 for outliers and value 1 for inliers. As an outlier, we consider a TDoA measurement that diverges from its ground truth value by more than ±0.5 [ms] (about ±5 sampling intervals). To eliminate the impact of the weighting scheme proposed in Section 3.1 for the heteroscedasticity of the measurements set, as it will be evaluated further on, in this experiment, all weights $d_{(i,k),j}$ are set to the value of 1. As a result, weights $w_{(i,k),j}$ are equal to 1 in the first iteration of the proposed Algorithm 2, and in the following iterations, the weights are set just to $b_{(i,k),j}$. For the purpose of this experiment, we set the reverberation time (${\mathrm{RT}}_{60}$) of the room to a mild value of 200 [ms], to achieve a small number of potential outliers that naturally occur in the TDoA measurement set. Next, a random inlier from the measurement set is distorted by changing its magnitude so that it becomes an outlier, which is realized by adding a random value sampled from a uniform distribution with an interval from 0.5 to 5.0 [ms]. The procedure of introducing TDoA measurement distortions is repeated until the desired (in this experiment) percentage of the outliers in the TDoA measurements is reached. The proposed outlier detection algorithm is evaluated on such a TDoA measurement set. This experiment was performed for TDoA measurement sets that contain from about 5% to 40% of the outliers, and the results of this experiment are depicted in Figure 5. By the detailed analysis of the results in Figure 5a, we can observe that for a very small number of outliers in the TDoA measurement set, the number of outliers detected by the proposed method is higher by about 5% than the number of outliers detected by the oracle outlier detector. On the other hand, with an increasing number of outliers in the measurement set, the number of outliers detected by the proposed method approximately approaches the value of the oracle detector. Such behavior can be well explained since when a small number of outliers are present in the measurement set, the standard deviation of residuals is going to be significantly smaller. As a result, the proposed thresholding rule identifies inliers with residuals significantly larger than the mean $\mu_{\tau }$ as outliers, even though these residuals are still much smaller than those of actual outliers. However, this small drawback should not substantially affect the quality of the solution in terms of the estimated parameters. On the other hand, when the number of outliers increases, which corresponds to the vast majority of real use cases, the proposed statistics and thresholding perform significantly better, and the proposed method nearly reaches the performance of the oracle detector. In order to confirm the correctness of outlier detection, in addition, we analyze the number of correctly detected outliers (true positive rate) and the number of inliers incorrectly detected as outliers (false positive rate). Figure 5b presents those two dependencies for a varying number of outliers in the TDoA measurement set. The results indicate that the proposed estimator correctly detects almost all outliers in the investigated range, with small variations of approximately 1%.

The second experiment evaluates the proposed weighting schemes that improve the estimation of the scaling factor and recording onset times when TDoA measurements are heteroscedastic due to the presence of the reverberation phenomenon. In Section 3.1, we propose the weighting scheme based on one of two types of distances, namely the maximum distance $\phi_{(i,k),j}^{\max}$ given by (12), and the average distance $\phi_{(i,k),j}^{\mathrm{avg}}$ given by (13). To carry out the experiment, we use the solution given by (11) to solve problem (6) with the two weighting schemes mentioned above and an unweighted version (i.e., no weighting is achieved by setting $w_{\left(i,k),j\right)}=1$), which can be considered equivalent to the baseline approach [29]. Parameter β in (14) is set to the value of 5 for this and all subsequent experiments. The evaluation is carried out for the reverberation time ranging from 150 to 750 ms with a 50 ms interval step. In order to quantitatively compare the aforementioned three different weighting options, we propose to apply two metrics, namely the mean relative absolute error (MRAE) and mean absolute error (MAE). The MRAE metric is used to evaluate the quality of the estimated scaling factor and is given by

(24) $$\mathrm{MRAE}\left(\gamma \right)=\frac{1}{K}\sum_{k=1}^{K}\frac{\left|{\hat{\gamma }}^{\left(k\right)}-\gamma^{\left(k\right)}\right|}{\left|\gamma^{\left(k\right)}\right|},$$

where the upper index ${\left(\;\cdot \;\right)}^{\left(k\right)}$ denotes the *k*th experimental trail. The relative error is used for the scaling factor since it depends on the estimated relative geometry determined in the first step, whose scale value depends on the initialization, i.e., for the initialization $I_{0}$, the ground truth scaling factor might be 10, but for the initialization $I_{1}\neq I_{0}$, the ground truth scaling factor might as well be equal to 100. For more details on this matter, the reader is referred to [29]. The MAE metric will be used for the recording onset times Δ, and it is given by

(25) $$\mathrm{MAE}\left(\Delta \right)=\frac{1}{KN}\sum_{k=1}^{K}\sum_{i=1}^{N}\left|{\hat{\delta }}_{i}^{\left(k\right)}-\delta_{i}^{\left(k\right)}\right|.$$

**Figure 6:**
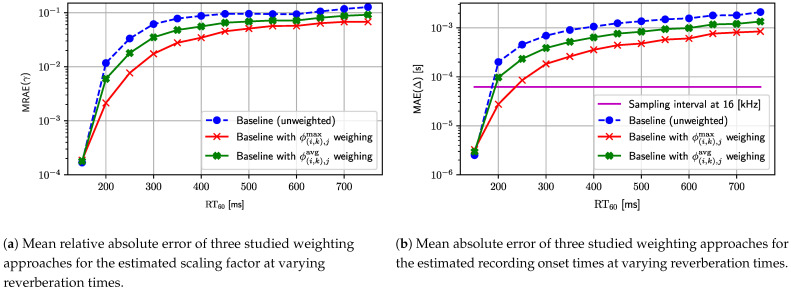
This figure presents the results of the experiment where three weighting schemes (maximal distance, average distance, and unweighted) are evaluated in the reverberant room. (**a**) Shows the mean relative absolute error of the scaling factor. (**b**) Shows the mean absolute error for the recording onset times. The reverberation time (${\mathrm{RT}}_{60}$) varies from 150 to 750 [ms].

Figure 6 shows the results of this experiment. The characteristic lines in both plots of Figure 6a,b exhibit similar relations and, thus, we discuss them both at the same time. All three weighting schemes achieve very similar (nearly the same) performance for the lowest reverberation time value (${\mathrm{RT}}_{60}=150$ [ms]), but a detailed inspection shows that the baseline achieves the best result, the next average distance weighting, and the maximum distance weighting. In these acoustic conditions, the number of outliers is very low (if they at all exist), and the distribution of measurements is almost homoscedastic. Consequently, it is expected that the baseline method (unweighted linear LS estimator) achieves the best results in nearly non-reverberant acoustic conditions. The situation changes completely when the reverberation time increases, as would be the case in any practical scenario. The difference between the three approaches becomes notable, the order is reversed, and the mutual relative gain stabilizes for all other tested reverberation times. As a result, the baseline method achieves the highest error in both considered error measures, the average distance weighting comes second, and the lowest error is observed for the maximal distance weighting. Regardless of the weighting scheme, both errors increase quickly with an increasing reverberation time level for the range between 150 and 400 ms, which is caused by the occurrence of an increasing number of outliers. For reverberation times greater than 400 [ms], the errors start to saturate. This is probably caused by a significant number of outliers in the measurements, which start to dominate during the model fitting. It should be noted that only the maximal weighting scheme is able to achieve average synchronization between devices lower than the sampling interval for the reverberation time set to 200 [ms]. Finally, since the maximal weighting scheme achieves the best results in most cases, this weighting is selected for all subsequent experiments as the default weighting scheme.

In the third experiment, similar to the previous one, several configurations of the proposed methods are compared for different reverberation time values, in the range from 150 to 750 [ms].

**Figure 7:**
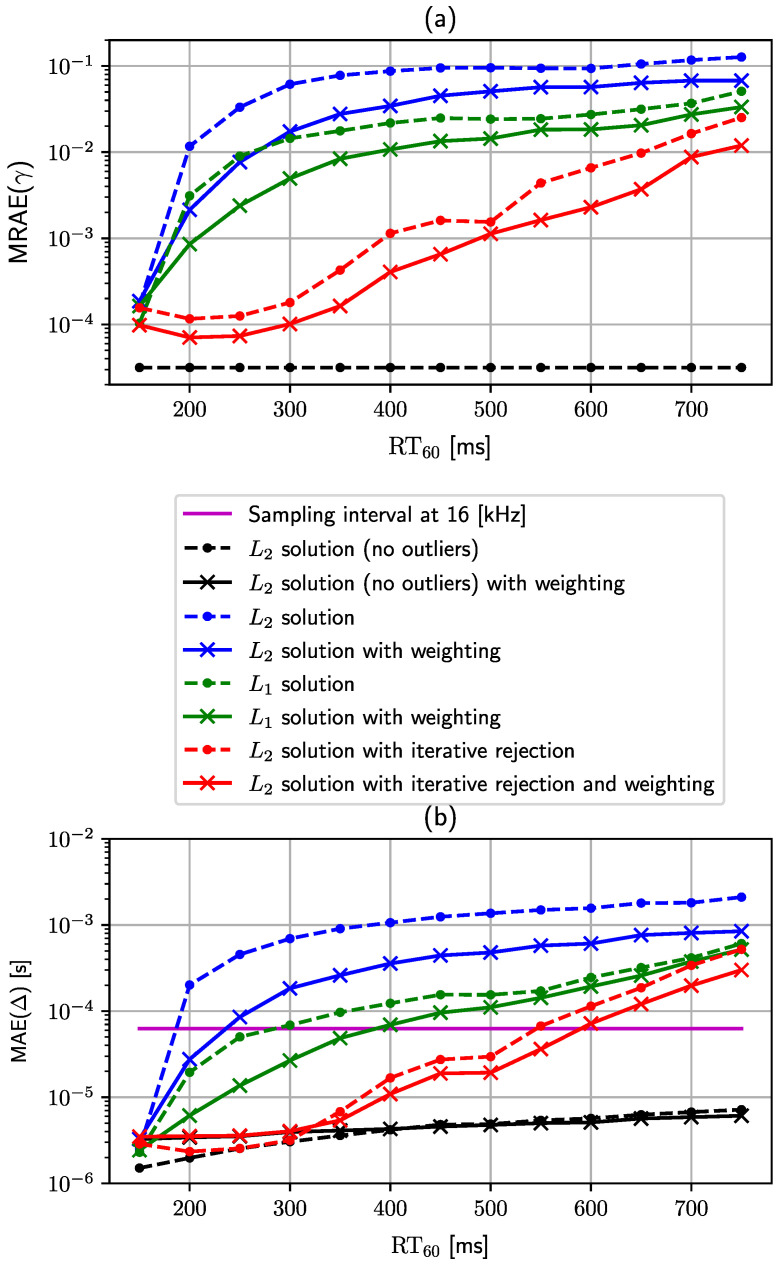
Comparison of the performance of the different configurations for varying reverberation times. (**a**) Mean relative absolute error of the scaling factor estimator. (**b**) Mean absolute error of the recording onset time estimator.

In the following, all investigated configurations are listed:

1. The first investigated configuration is a baseline, but instead of using a reverberant and noisy microphone signal for the TDoA estimation, only the direct propagation path with the microphone noise is taken into account. Such configurations will provide a lower reference bound on estimation errors for other configurations. The results for this configuration are denoted by a black dashed line with a dot marker in Figure 7.
2. The second configuration is the baseline approach, in which the LS estimator with all weights set to 1, i.e., $w_{(i,k),j}=1$. The results for this configuration are denoted by a blue dashed line with a dot marker in Figure 7.
3. The third configuration is the LS estimator with maximal distance weighting $w_{(i,k),j}=\phi_{(i,k),j}^{\max}$. The results for this configuration are denoted by a blue solid line with a cross marker in Figure 7.
4. The next configuration will be an LA estimator with all weights set to 1 $w_{(i,k),j}=1$ . The results for this configuration are indicated by a green dashed line with a dot marker in Figure 7.
5. The fifth configuration is an LA estimator with maximal distance weighting $w_{(i,k),j}=\phi_{(i,k),j}^{\max}$. The results for this configuration are denoted by a solid green line with a cross marker in Figure 7.
6. The sixth configuration is an approach proposed in Algorithm 2 with one change, all weights $d_{(i,k),j}$ are set to 1. The results of this configuration are denoted by a red dashed line with a point marker in Figure 7.
7. Finally, the last configuration is an approach proposed in Algorithm 2, with the maximal distance weighting $w_{(i,k),j}=\phi_{(i,k),j}^{\max}$. The results for this configuration are denoted by a solid red line with a cross marker in Figure 7.

**Figure 8:**
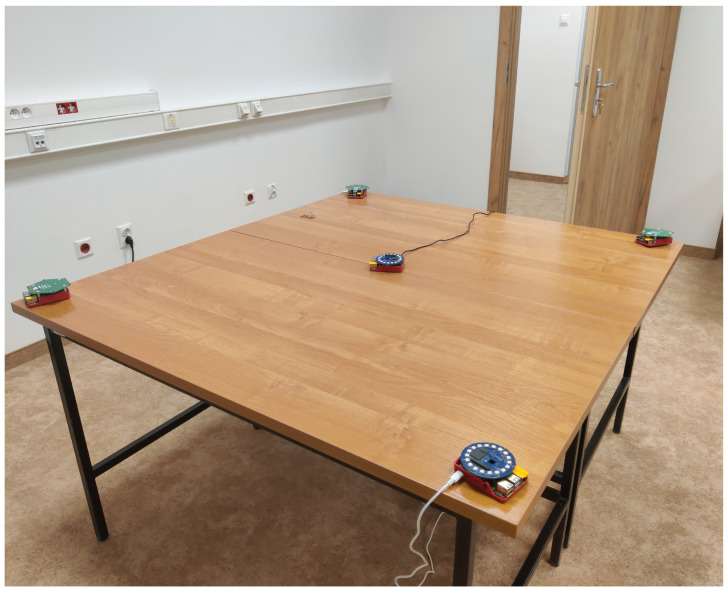
Five microphone arrays placed on the table in AGH’s Audio Lab room.

The first reference baseline configuration exhibits constant values of both metrics due to its independence from the reverberation time. The MRAE value for the scaling factor settles at a level approximately equal to $2\times 10^{-4}$, and the constant value of MAE for the recording onset times is approximately $1\times 10^{-6}$ [s]. These values set an experimental lower bound on the estimation errors for the evaluated results presented in Figure 8. In general, all three configurations based on the weighting with the proposed maximal distance weighting scheme, i.e., configurations 3, 5, and 7, show performance improvement in their respective unweighted versions, i.e., configurations 2, 4, and 6. The only test point where the difference is not significant between all configurations is when there is almost no reverberation, i.e., for the lowest reverberation time value of 150 [ms]. However, from Figure 8a, it can be seen that configurations 4 and 7 still achieve a slightly better result for the scaling factor. There is also a substantial difference in performance between the LS, LA, and the proposed LS with iterative rejection. In most test cases, a simple LS estimator (configurations 2 and 3) reaches the highest error value, as it is most exposed and sensitive to outliers. Intermediate performance is obtained for the LA estimator (configurations 4 and 5), which is less sensitive to the outliers and achieves much lower estimation errors than the simple LS estimator. Finally, the proposed method summarized by Algorithm 2 (configurations 6 and 7) combines the assets of the LS and LA estimators and achieves overall remarkably better results than other methods. A significant advantage of the proposed method is especially visible for the mild to medium/high reverberation time (ranging from 200 [ms] to 500 [ms]), where the difference between the baseline and the proposed method in terms of the estimation error is close to three orders of magnitude at some test points. Furthermore, the proposed method (configuration 7) reaches nearly the lower bound for test points from 200 [ms] to 500 [ms] as it is close to the performance of the reference configuration 1 for that range. With an increasing reverberation time (above 600 [ms]), all configurations are starting to approach similar levels of error but still keep performance order intact. Lastly, the synchronization performance of the proposed method allows the WASN to be synchronized to the level below a single sampling interval for a significantly higher reverberation time than in the baseline method. In this experiment, the cutoff reverberation time for the baseline method is 200 [ms]. The weighted LS (configuration 3) improves it roughly by about 50 to 250 [ms]. The LA estimators achieve a slightly better cutoff reverberation time (configurations 4 and 5), which reaches values of 300 [ms] and 400 [ms], respectively. Finally, a massive improvement in terms of the cutoff reverberation time is achieved by the proposed method (configuration 7), which pushes its value by another 200 [ms] to the level of 600 [ms].

Up to this point, we evaluated the proposed method under conditions that mitigate the impact of the estimation of the relative geometry (which is performed within the first optimization step) on the result of the second optimization step (which constitutes the major issue addressed in this article). Recall that this mitigation was achieved, in previous experiments, by using DoA measurements estimated with only a noisy microphone signal (without reverberation). To fully evaluate the performance of the proposed approach in practical deployments, we present the results of two additional experiments that do not take into account this mitigation, in which reverberant microphone signals are used for DoA estimation too. In the following two experiments, we compare the proposed method with several well-established state-of-the-art approaches [20,28,31]. To this end, we introduce three additional evaluation metrics that show the average displacement of the estimated positions from the ground-truth values. These metrics are defined as

(26a) $$\mathrm{RMSE}\left(\hat{\Theta }\right)=\frac{1}{KN}\sum_{k=1}^{K}\sum_{i=1}^{N}{∥{\hat{\theta }}_{i}-\theta_{i}∥}^{2},$$

(26b) $$\mathrm{RMSE}\left(\hat{N}\right)=\frac{1}{KN}\sum_{k=1}^{K}\sum_{i=1}^{N}{∥{\hat{n}}_{i}-n_{i}∥}^{2},$$

(26c) $$\mathrm{RMSE}\left(\hat{S}\right)=\frac{1}{KS}\sum_{k=1}^{K}\sum_{j=1}^{S}{∥{\hat{s}}_{j}-s_{j}∥}^{2}.$$

Table 1 presents the results of experiment 4, which are obtained with the setup described at the beginning of this section with numerically simulated microphone signals. Both TDoA and DoA were estimated from reverberant noisy microphone signals. Analyzing the numerical results from Table 1, it can be observed that approaches such as in [28] do not take into account the compensation for the onset time, and do not achieve reasonably low position and orientation errors, regardless of the room reverberation time level. In the case of the moderate reverberation time (${\mathrm{RT}}_{60}=400$ [ms]), the other two methods [20,31] and the approach proposed in this article achieve very comparable results. In the case of a long reverberation time, the methods presented in [20,31] again perform comparably, while the algorithm proposed in this article achieves significantly better results for all evaluation metrics. The following conclusions can be drawn from these results. In the case of long reverberation time levels, the proposed method is able to correctly identify and reject outliers despite the non-ideal relative geometry, estimated in the first step of the network calibration algorithm, and as a consequence, it yields much better estimates of the unknown parameters of the wireless acoustic sensor network. On the other hand, the proposed algorithm does not significantly deteriorate the results for moderate reverberation time levels compared with other existing methods and, in fact, it achieves similar results under moderate conditions.

**Figure 9:**
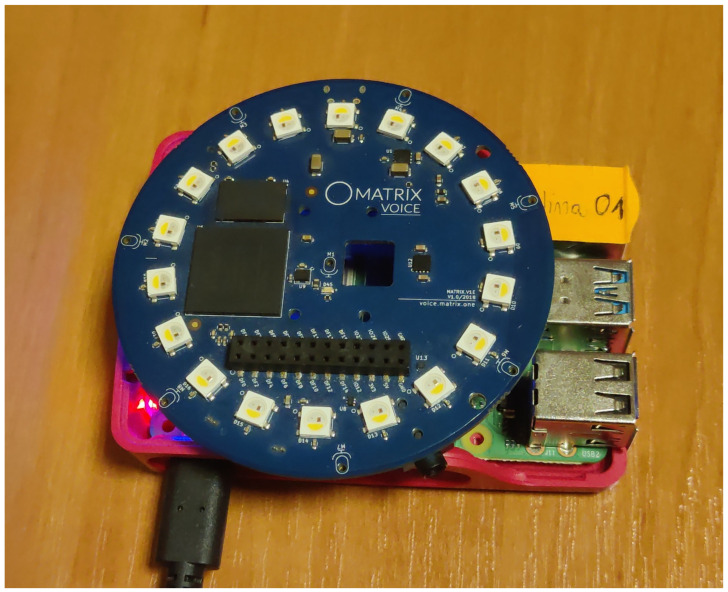
A circular microphone array, with eight microphones, mounted on a single-board computer with a wireless network interface.

As the final experiment, an evaluation was performed using acoustic signals measured using real distributed microphone arrays connected by a wireless network in the real Audio Lab room at the premises of AGH University of Krakow (the setup is shown in Figure 8). In experiment 5, we use five circular microphone arrays, each containing eight microphones, mounted on a single-board computer with a Wi-Fi interface, as depicted in Figure 9.

The microphone arrays were placed on a table, while a person walked through the room and spoke short sentences in different locations across the room. A total of 10 such speaker locations were used as the source signals (used for the calibration of the distributed network). Table 2 presents the numerical results obtained after processing the acoustic signals with the proposed algorithm and three baseline methods [20,28,31]. The conclusions, made from observations of the results obtained from real measured acoustic signals, coincide well with the conclusion of experiment 4 for synthetic signals. For medium room reverberation conditions, the errors in position and orientation are slightly smaller for the proposed algorithm than for the remaining three existing methods. These results confirm that the proposed approach actually notably improves the precision of all studied estimates.

In summary, the proposed algorithm substantially outperforms the baseline approach across a broad spectrum of reverberation time values. Unlike the current state-of-the-art methods, our proposed method (i.e., Algorithm 2) robustly and accurately estimates both the geometry scaling factor and the recording onset time values, even under realistic room reverberation conditions.

## 5. Conclusions

This article addresses the problem of self-localization and synchronization of distributed microphone arrays in reverberant rooms using a popular two-step approach. The primary focus is on the second step, in which the scaling factor and recording onset times are estimated using TDoA measurements. We analyze the problems of TDoA estimation in the presence of reverberation and the implications these errors have on the estimation of the absolute geometry and synchronization of the onset times between the distributed arrays, both of which occur in the second step.

In the context of estimating the required scaling factor and recording onset times, this analysis leads to identifying two major issues. The first one is the heteroscedasticity of the TDoA measurement set, and the second concerns the occurrence of outliers. Both of these issues relevantly impact the state-of-the-art methods, which are optimal for Gaussian noise. In order to mitigate heteroscedasticity, this article proposes a weighted linear least squares estimator supported by two weighting schemes that exploit the relative geometry estimated in the first step, as well as domain knowledge presented in Section 3. To address the problem of outliers, we propose an iterative TDoA reweighing algorithm with binary weights, which exploits least absolute value minimization and thresholding to detect and reject strong outliers in TDoA measurements. The experimental evaluation of the proposed method shows a significant improvement in accuracy and robustness to adverse acoustic conditions caused by room reverberation.

## Figures and Tables

**Table 1:** Comparison of the proposed method with alternative approaches for self-calibration. RMSE of the estimated parameters for K=100 random geometries in a simulated reverberant room of size 10×10×3 [m] with different reverberation time levels (${\mathrm{RT}}_{60}=400$ and 800 ms).

Method	${\mathrm{RT}}_{60}$	$\mathrm{RMSE}(\hat{\Theta })$	$\mathrm{RMSE}(\hat{N})$	$\mathrm{RMSE}(\hat{S})$	$\mathrm{MAE}(\hat{\Delta })$
[28]	400 [ms]	28.9	2.52 [m]	3.26 [m]	13.76 [ms]
	800 [ms]	38.5	2.99 [m]	4.23 [m]	14.76 [ms]
[31]	400 [ms]	1.7	0.11 [m]	0.14 [m]	1.13 [ms]
	800 [ms]	7.1	0.27 [m]	0.41 [m]	2.76 [ms]
[20]	400 [ms]	2.8	0.14 [m]	0.16 [m]	1.16 [ms]
	800 [ms]	8.0	0.29 [m]	0.39 [m]	2.66 [ms]
Proposed	400 [ms]	3.1	0.13 [m]	0.17 [m]	1.16 [ms]
	800 [ms]	5.1	0.18 [m]	0.25 [m]	1.36 [ms]

**Table 2:** Comparison of the results obtained from the proposed method and alternative state-of-the-art approaches for self-calibration performed with acoustic microphone signals recorded in the real room. At the time of publication, we were unable to measure the compensation for the recording onset times due to hardware limitations; thus, the table lacks a column for $\mathrm{MAE}(\hat{\Delta })$.

Method	$\mathrm{RMSE}(\hat{\Theta })$	$\mathrm{RMSE}(\hat{N})$	$\mathrm{RMSE}(\hat{S})$
[28]	0.43	0.99 [m]	1.23 [m]
[31]	0.15	0.21 [m]	0.33 [m]
[20]	0.12	0.22 [m]	0.31 [m]
Proposed	0.08	0.14 [m]	0.20 [m]

## Data Availability

Data are contained within the article.

## Abbreviations

The following abbreviations are used in this manuscript:

WASN	wireless acoustic sensor network
ToF	time of flight
ToA	time of arrival
TDoA	time difference of arrival
DoA	direction of arrival
RANSAC	random sample consensus
TOPS	test of orthogonality of projected subspaces
SRP-PHAT	steered response power with phase transform
GCC-PHAT	generalized cross-correlation with phase transform
vMF	von Mises–Fisher
PDF	probability density function
LS	least squares
LA	least absolute
ML	maximum likelihood
